# Health Technology Assessment for In Silico Medicine: Social, Ethical and Legal Aspects

**DOI:** 10.3390/ijerph19031510

**Published:** 2022-01-28

**Authors:** Carlo Giacomo Leo, Maria Rosaria Tumolo, Saverio Sabina, Riccardo Colella, Virginia Recchia, Giuseppe Ponzini, Dimitrios Ioannis Fotiadis, Antonella Bodini, Pierpaolo Mincarone

**Affiliations:** 1 Institute of Clinical Physiology, National Research Council, 73100 Lecce, Italy; leo@ifc.cnr.it (C.G.L.); mariarosaria.tumolo@unisalento.it (M.R.T.); recchia@ifc.cnr.it (V.R.); 2 Department of Biological and Environmental Sciences and Technology, University of Salento, 73100 Lecce, Italy; riccardo.colella@unisalento.it; 3 Institute for Research on Population and Social Policies, National Research Council, 72100 Brindisi, Italy; giuseppe.ponzini@cnr.it (G.P.); pierpaolo.mincarone@irpps.cnr.it (P.M.); 4 Unit of Medical Technology and Intelligent Information Systems, Department of Materials Science and Engineering, University of Ioannina, 45110 Ioannina, Greece; fotiadis@uoi.gr; 5 Department of Biomedical Research, Institute of Molecular Biology and Biotechnology—Foundation for Research and Technology Hellas (IMBB-FORTH), 45115 Ioannina, Greece; 6 Institute for Applied Mathematics and Information Technologies “E. Magenes”, National Research Council, 20133 Milan, Italy; antonella.bodini@mi.imati.cnr.it

**Keywords:** in silico medicine, digital twin technology, artificial intelligence, computer modeling and simulation, ethical aspects, legal aspects, social aspects, health technology assessment

## Abstract

The application of in silico medicine is constantly growing in the prevention, diagnosis, and treatment of diseases. These technologies allow us to support medical decisions and self-management and reduce, refine, and partially replace real studies of medical technologies. In silico medicine may challenge some key principles: transparency and fairness of data usage; data privacy and protection across platforms and systems; data availability and quality; data integration and interoperability; intellectual property; data sharing; equal accessibility for persons and populations. Several social, ethical, and legal issues may consequently arise from its adoption. In this work, we provide an overview of these issues along with some practical suggestions for their assessment from a health technology assessment perspective. We performed a narrative review with a search on MEDLINE/Pubmed, ISI Web of Knowledge, Scopus, and Google Scholar. The following key aspects emerge as general reflections with an impact on the operational level: cultural resistance, level of expertise of users, degree of patient involvement, infrastructural requirements, risks for health, respect of several patients’ rights, potential discriminations for access and use of the technology, and intellectual property of innovations. Our analysis shows that several challenges still need to be debated to allow in silico medicine to express all its potential in healthcare processes.

## 1. Introduction

Over the past decade, computer modeling and simulation (CM&S) technologies have increasingly been applied in disease prevention, diagnosis and treatment by simulating real biological processes in a virtual environment [1]. These technologies, also referred as in silico medicine or computational medicine, can, with varying levels of autonomy, make predictions, recommendations, or decisions influencing real or virtual environments [2] and have four targets: (a) doctors, through patient-specific models to support medical decisions within the precision medicine paradigm (digital twins or digital avatars, when a dynamic pairing is done with the modeled physical entity); (b) citizens, through easier and more pervasive access to their personal data—including those collected by wearable and environmental sensors—and personalized health status forecasting, providing advice for self-management; (c) decision-makers, by modeling person-to-person interactions and factors affecting health at population level (e.g., to prevent and manage epidemics) within the precision public health paradigm; and (d) research organizations/companies, through modeling virtual patient populations applied to reduce, refine, and partially replace pre-clinical and clinical assessment of medical technologies [3,4,5]. Two kinds of models can be used and also combined in hybrid solutions: (a) mechanistic models that incorporate scientific knowledge in biophysics, biochemistry, and physiology and are based on cause-effect relationships, and (b) phenomenological models that start from sufficiently numerous empirical observations and use statistics, system identification methods, or artificial intelligence (AI) to develop predictors without any causal assumption [6]. CM&S solutions can also be a combination of technologies. For example, digital twins can integrate modern technologies such as smart sensors (also as an application of the Internet of Things), data analytics, and AI to embed learning, intelligence, and autonomy [5,7,8].

In silico medicine has the potential to revolutionize the future of healthcare and is among the recognized emerging health technologies [9]. Its market is expected to grow rapidly in the next few years. The global digital-twin market size, valued at USD 3.1 billion in 2020, is expected to reach USD 48.2 billion by 2026, with an increasing demand for applications in the healthcare and pharmaceutical sector due to the outbreak of the COVID-19 pandemic [10]. With specific reference to drug discovery, the global in silico market was valued at USD 2.09 billion in 2018 and is anticipated to grow to over USD 7.92 billion by 2029 [11]. The interest of pharmaceutical companies stems from the opportunity to increase the currently low clinical trial success rates (for US trials, respectively, under 14%, 36%, 59%, and 86% for Phase 1, Phase 2, Phase 3, and Food and Drug Administration submissions for approval) by adopting CM&S [12].

In silico medicine requires or supports several important changes [13]. Healthcare workers need to adapt their clinical practice and update their expertise to communicate risks, make predictions, and discuss trade-offs with patients. Patients should acquire a more active role in managing their own health. Healthcare managers can more easily implement home care plans as an alternative to hospital care whenever possible. These changes may challenge some key principles [13,14,15]: transparency and fairness of data usage; respect of data privacy and protection across platforms and systems; guarantee of data availability and quality; respect for intellectual property; data reusability; equal accessibility for persons and populations.

Several social, ethical, and legal issues, not present in more traditional technologies such as drugs, image-guided procedures, and implantable devices, may consequently derive from in silico medicine and should be duly considered for a comprehensive understanding of its value in order to support decision-makers who deliberate on its adoption. Although introduced well before the advent of in silico medicine [16], health technology assessments (HTAs) can be embraced for this purpose as a multidisciplinary process that summarizes information from several domains, including those focused on in this work, in addition to efficacy, safety, economic, and organizational issues related to the use of health technology in a systematic, transparent, unbiased, and robust manner [17,18]. Furthermore, the COVID-19 pandemic has had a dramatic impact on all facets of in-person care, accelerating the acceptance of technology-mediated care (e.g., telehealth or hybrid care models) [19,20]. The possibilities offered by CM&S also support the continuation of drug development and clinical research by allowing the restrictions related to COVID-19 to be overcome [21]. The pandemic has made it even more urgent to discuss and assess the impact on society of adopting in silico solutions.

In this narrative review, we wish to provide an overview of the social, ethical, and legal impact of in silico medicine along with some practical suggestions for assessing these domains in light of the opportunities that HTAs can provide to deal with them.

## 2. Materials and Methods

To grasp the complexity of the problems faced, we performed a narrative review, as also suggested by Greenhalgh and colleagues [22]. A narrative approach was preferred to a systematic one since we were not interested in reporting the data collected on specific cases but in extrapolating problems. We have limited our search to works published in English. The review of the literature was carried out without other restrictions: we included all years (1900–2021, December) and all document types (e.g., journal articles, books, chapters, conference proceedings, websites of relevant organizations). The search was conducted on MEDLINE/Pubmed, ISI Web of Knowledge, Scopus, and Google Scholar by a multidisciplinary team of socio-economists, methodologists, and biomedical engineers. We combined the concept of technology of interest with the target HTA domains (social, ethical, and legal) using the AND operator. Technologies have been identified through the following keywords: in silico medicine/trial, digital twin technology, hypermodel, computer modeling/simulation, in silico modeling, patient modeling, personalized medicine, artificial intelligence, Big Data, machine learning OR decision support system. HTA domains of interest were identified by the following terms: HTA, ethics, legal, social, acceptance, health literacy, public health, equity, patient–clinician relationship, usability, access, engagement, social justice/responsibility, informed consent, data protection, healthcare delivery, good practice OR regulation. Studies were assessed through visual inspection of titles and abstracts and were included when discussing the social, ethical, and legal implications of in silico medicine or suggesting operational solutions to assess these domains in HTA reports. Forward citation of relevant papers was also adopted to increase the sensitivity of the search process. The evidence collected on the three domains was analyzed by all the authors in order to identify the most relevant concepts. CGL, MRT, AB, SS, and PM produced the synthesis that was subsequently discussed by all authors and reported in this work.

## 3. Results

In total, we considered 26 review papers (including editorials, comments, and viewpoints), 13 research studies, 9 reports from international organizations or research consortia, and 2 official documents (laws, recommendations). All the included works are reported in the references.

In the following two sections, we discuss the results of our research.

### 3.1. Social Issues of In Silico Medicine

Regarding the social domain, the broad socioeconomic context of the pharmaceutical and biomedical sector in which in silico medicine is applied must be considered. Public healthcare budgets appear to be increasingly less able to keep up with the pace of healthcare expenditure [23]. This requires the development of policies capable of simultaneously pursuing three potentially conflicting objectives: (1) timely and equitable access for patients to high-quality health services, (2) the financial sustainability of publicly funded health systems, and (3) the valorization of industrial innovation [24]. The trade-off among these goals should not be found through a downsizing approach with spending cuts that truncate demand or sacrifice technology improvements. Indeed, in the long run, these strategies may undermine the provision of equitable and comprehensive healthcare for the population or some social groups without sufficient purchasing power [25]. Rather, in silico medicine is a promising approach to reduce the above-reported tensions. Several research groups are working to evaluate the capacity of the models to represent reality and to assess their impact on healthcare costs and on the duration of pre-clinical and clinical development [5,26,27].

While technicians are working hard to design, develop, and test in silico applications for healthcare, stakeholders are well aware that several cultural and infrastructural issues need to be addressed for a full uptake of in silico medicine and, more generally, of digital technologies [28,29]. In terms of cultural issues, there can be hesitancy and cultural resistance to the reliability of simulated scenarios due to the stigma of the unreal [30]. Another critical issue for healthcare personnel could be the level of expertise when asked to use CM&S-based systems, which may require a long training period. This may have a negative impact on the clinical effectiveness of CM&S, especially in the initial period [31,32]. For applications aimed directly at citizens, the level of knowledge and literacy required should also be considered so as not to create inequities [33]. To date, few studies have considered the effectiveness of digital solutions in marginalized populations (by race and ethnicity, socioeconomic status, language, digital literacy and numeracy, health literacy, frailty) for whom the potential reduction of real human contact can have negative consequences [34,35].

CM&S-based applications require important digital infrastructure (e.g., fast communication networks and high computing power and storage capacity) that is not available everywhere and for every intended user, thus compromising a public health approach to the use of digital technology [33,36]. This aspect should be analyzed as part of the equity issues considered in the social impact of a proposed technology.

Several methods are proposed to gather the necessary evidence to assess the social domain of the HTA for a specific technology: (a) assessment of existing literature, preferably through systematic methods; in case the central questions cannot be answered on the basis of existing studies, (b) new primary studies involving citizens (individually or as associations) and experts of the domain (primarily, physicians and sociologists) with a quantitative (by means of surveys) or qualitative approach (through interviews, focus groups, discussion forum, and roundtables with experts); (c) analysis of the technical documentation provided by the manufacturer/supplier of the technology; (d) analysis of the regulations [37,38].

The choice of methodologies to be applied is framed by the specific question to be addressed. In Table 1, we propose some practical examples of questions derived from the above-reported discussion with our suggestion of the methods, among those listed above, that can help to generate the required evidence.

As indicated by the European Network for Health Technology Assessment (EunetHTA), advanced skills in social science are required to perform HTA analyses in the social domain [17]. Among the expertise reported, we believe the following are particularly useful to perform the analyses proposed in Table 1: communication science, health services research, health sociology, medical decision-making, medical ethics, medical sociology, and science and technology studies. Of course, competencies in systematic reviews and meta-analyses and statistics are also recommended for quantitative studies.

### 3.2. Ethical and Legal Issues of In Silico Medicine

Ethical and legal issues are often intertwined due to the necessity of protecting ethical principles and values through norms. For this reason, we have chosen to address these aspects in a single section.

In line with a cultural vision of science and technology as democratized territories, systemic changes need a dialog among stakeholders on their predicted ethical impact [39]. Early identification and evaluation of ethical issues can help our contemporary society to be better prepared for future moral dilemmas and can also guide research and development or usage practices so as to avoid or minimize ethically undesirable consequences [40].

One of the key questions when addressing the ethical assessment of technology is the tension between expected advantages (for example, the social value of improving the quantification of human health and sickness, the personalization of care, and the awareness of individuals) and unintended consequences, also considering that the success and consequent wide adoption of a technology can create new areas of ethical risks [35,41].

This section discusses some ethical implications that are worth addressing in HTAs.

When CM&S is applied to the research and development process of a medical technology, the possibility of reducing the sample size or totally avoiding the use of animals in tests and/or human participation in trials is an unquestionable value. Even shorter times to estimate long-term effects can be helpful in focusing on the most promising solutions and reducing the time to market. When CM&S is adopted in clinical practice, for example, to estimate the presence or prognosis of a pathology or to define the best treatment for a specific subject based on a simulation, it is very important to have a clear idea of the impact of potential errors of the simulation tool and of any limitations for the quality of clinician interpretations used during training [42]. In the case of screening applications, the choice to prefer accuracy, sensitivity, or specificity (for example, when choosing a particular threshold to guide decisions) may also have ethical implications: the chances of survival can significantly decrease for a delayed diagnosis, and invasive or ionizing procedures may be an unnecessary additional risk [17,43].

When CM&S is applied in clinical practice, shared and fully aware decisions require the interaction of three actors as CM&S becomes something like a third agent in the patient–physician relationship. In silico models could adopt algorithms for data analysis that may not meet the transparency standards of evidence-based medicine (a phenomenon referred to as black-box medicine) [44], and information could hardly be conveyed in a consistent manner with the patient’s language of preference and culture [45]. Therefore, new information asymmetries could arise between physician and patient with respect to CM&S [46]. In this context, it is important to emphasize that information asymmetry involves (directly and reciprocally) both the physician and the patient and that the “third agent” (i.e., CM&S) can take on the role of a sort of stone guest. From an HTA perspective, a number of measures can be considered. The degree of transparency in data collection should be evaluated and discussed together with potential sources of bias and ways to reduce them [47]. The presence of an adequate time for discussion between the patient and clinician (to examine the benefits and risks of a treatment, the potential alternatives, the strength of predicted risks and prognostic outcomes, and to consider the patient’s right not-to-know and its consequences) should be assessed [48,49].

In silico medicine, as an emerging technology, can be either not accessible to everyone or not covered by health insurance for everyone, thus contributing to widening gaps that already exist based on socioeconomic status [35]. The context of the application should, therefore, be framed to identify possible sources of inequalities.

Another element that can raise ethical concerns is the aging of the technology: continuous iterative innovations can lead to rapid obsolescence, with a subtle pressure for the not necessarily urgent purchase of the latest generation technologies, distracting resources from more relevant issues. A list of versions and key changes should be available to understand the product’s development and consciously manage upgrading evidence [50]. This is an element of value as it favors a conscious comparison among the evidence generated with new releases of the assessed technology.

One of the pillars of in silico medicine is the capacity to use Big Data, intended as patient-specific data from various sources, such as genomics, which integrates a multiplicity of heterogeneous data with the unavoidable risks and challenges associated with access and sharing [51,52]. Furthermore, these integrations create an ethical dilemma for the clash between public and individual ethical principles: the responsibility of improving community health and that of protecting patient confidentiality [53]. For the use of Big Data, several other ethical issues, sometimes with consequent legal implications, concerning patients’ autonomy can arise for:

A. The informed consent—three elements have been highlighted by Andreotta and colleagues [54]:
  - The transparency (or explanation) problem due to the reluctance to disclose the functioning of algorithmic reasoning that opposes the statutory right to an explanation on the use of data; far from being an exhaustive explanation of technical aspects that, on the contrary, could overwhelm the subjects, transparency should clarify the context and the potential harm caused by a decision.
  - The re-purposed data problem—new AI algorithms can be applied to existing data sets to generate new information for different purposes, and this could make the original consent no longer applicable. Proper authorization should be collected for the potential future use of data for research purposes (a vital necessity for in silico medicine). Data collection is often retrospective, and obtaining explicit authorization for new purposes may not be easy or feasible: in Europe, for example, the data protection regulation requires clear identification of the purpose of the processing at the time of collection, and this can compromise the use of retrospective data [55].
  - The meaningful alternatives problem—this arises when users are not given alternative choices if they do not wish to consent.

New approaches to informed consent are suggested. The use of dynamic consent models offers individuals the possibility to change their preferences over time to recalibrate their decision on new uses of their data beyond what initially permitted [56,57]. Comic or pictorial descriptions can be integrated into consent forms and, since they simplify complex concepts, facilitate communication among patients and experts [54].

B. The protection of individual citizens from the harmful use, also due to security breaches, of their personal data (e.g., social stigma, screening in insurance contracts, and discrimination on the labor market). In this regard, as shown in an interesting way by Rocher and colleagues, de-identification could be insufficient to ensure anonymization [58]. They found that 99.98% of Americans would be correctly re-identified in any dataset using 15 demographic attributes. A new approach to solving the challenge surrounding big health data sharing is the generation of virtual (synthetic) data created from real data, which, compared to anonymized data, protect privacy by adding statistically similar information, thus preserving the possibility to draw valid statistical inferences [59]. Virtual data reduce legal constraints when using sensitive or other types of regulated data and tailor the data needs to certain conditions that are difficult to achieve with authentic data.
C. The need for harmonized data-sharing systems—this also implies the standardization of data formatting. This is a major challenge not only for high-income countries but also for low- and middle-income economies [60]. The adoption of common transnational regulations and standards can help solve this problem as well as support innovation [61].
D. Equity in access—even in silico medicine, like all the other cases of digitization of healthcare, can harm health equity: [57] it is developed with homogeneous, highly educated, and advantaged populations in mind [33]. In this case, a solution can be the adoption of universal design approaches, as defined by the Center for Universal Design at North Carolina State University, i.e., the design and implementation of a technology in order to allow users to access, understand, and use it regardless of their abilities [62]. Another issue of equity may be generated in case of misestimated risks for groups (minorities) that are underrepresented in the derivation phase [63]. Biases in algorithm definition and poor training of analysts may pose risks to equity [57]. These critical points should be verified in the methodological design of derivation studies.

The above-reported issues imply the necessity to include an assessment of data management and transparency in HTA reports.

A further possible ethical issue regarding the equitable use of public resources is linked to the intellectual property of innovations based on public funds. The support provided to companies by public institutions could be planned in advance under agreements that consider future compensations (for example, through royalties) [64]. As regards access to patented knowledge generated with the support of public funds, companies could allow it for research purposes. The transparency, reproducibility, and reusability of research data are also key elements, especially in the case of publicly funded research [15]. These elements of social compensation could also be included in the value assessment of technologies, especially when adopting a societal perspective. A systematic appreciation of the ethical responsibility underlying these models of reduced profitability could give strategic orientations of corporate social responsibility a competitive advantage, with positive repercussions for society [64,65,66].

A critical legal issue is, by definition, the process for the marketing authorization of technologies assessed through in silico methodologies. While current regulations do not allow simulations to support applications for United States Food & Drug Administration (FDA) approval and the European CE marking of a medical device, both the FDA and the European Medicines Agency (EMA) are working on this issue. FDA scientists are discussing how to use in silico data to fill knowledge gaps of clinical trials, and, specifically, their current efforts are devoted to establishing good practice guidelines for simulations [67]. In Europe, the EMA recognizes in its proposed strategy on Regulatory Science to 2025 the importance of in silico methods almost exclusively in association with the reduction in animal testing [68]. A discussion is currently ongoing among stakeholders on potential applications for the reduction, refinement, and, in some cases, even replacement of clinical trials [68]. The European Economic and Social Committee has specifically called for the creation of Good Simulation Practices [69]. Notably, among the proposals, there is a recommendation to the Commission for developing Good Simulation Practices to provide industries with a “definite regulatory framework to guide pre-clinical activity and in clinical trials through CM&S in healthcare”.

An assessment of ethical and, therefore, legal issues is also suggested through the participation of key stakeholder groups (including end-users) to grasp the wide range of ways in which a technology can interact with the environment and social institutions to broaden the perspectives of the evaluation and to achieve broad legitimacy for its adoption [70].

A suggested approach for the study of ethics in emerging technology is Brey’s anticipatory technology ethics [40]. In this approach, a forecasting analysis is also recommended to consider possible harms, violation of rights, and negative impacts on well-being and distributions of goods, resulting from how a technology is likely to evolve over time, how it could be combined with other technologies, and how it could be used for new purposes.

As in the section on social issues, in Table 2, we propose some practical examples of questions and the associated suggested method.

## 4. Conclusions

At the beginning of the 1990s, where we can roughly place the introduction of the term, in silico meant “performed on computer or via computer simulation”. Nowadays, thanks to the impressive technological and methodological development of computer science and data analysis, the term includes any experimental technique performed by computers. By integrating biological and medical data from many different sources, computational models, simulations, and visualization techniques are developed with the aim of ultimately promoting breakthroughs or advances in medicine and therapy.

In silico medicine is generally recognized as a powerful tool that includes numerous benefits, such as the possibility to move towards personalized medicine (identification of health conditions, prognosis estimation, and support for identifying the best expected treatment), the reduction of animals’ and patients’ involvement in trials, the reduction of research and development costs, and the acceleration of time-to-market.

A good analysis of in silico technologies requires comprehensive information on a wide range of issues that reflect what is likely to happen in a health and social system. In fact, the ongoing process of industrialization of in silico medicine and its reach into society necessarily raises several social, ethical, and legal issues that should be duly considered. Health technologies have long been studied for safety, effectiveness, cost and other traditional concerns. Since its systematization in the 1960s, the development of the technology assessment has found a field of application and inspiration for development in precisely the health sector due to the growing public interest raised by the introduction of some health technologies (e.g., contraceptives, artificial organs) in matters that go far beyond immediate health effects. HTA, as a multidisciplinary and structured analysis performed for the purpose of providing input to a political decision, has the potential of evolving and enlarging its applications to answer all the issues raised by in silico technologies and not just those specific to health.

In this paper, we provide an overview of the social, ethical, and legal impact of in silico medicine and present a discussion on how HTAs can deal with these domains.

To grasp the complexity of the problems faced, we have performed a narrative review of the literature and included journal articles, books, chapters, conference proceedings, and websites of relevant organizations discussing the social, ethical, and legal implications of in silico medicine and suggested operational solutions to assess these domains in HTA reports. Our principal aim was to provide an overview of the social, ethical, and legal impact of in silico medicine, also trying to operationally translate the discussed points into practical examples of questions, suggested methods, and required expertise.

The following key aspects have emerged as general reflections with an impact at an operational level: the evolving scenario in the regulatory domain when CM&S is adopted to support research and development processes, the cultural resistance to the whole concept of computer simulation, the approach to enable the effective participation of patients and stakeholders in the decision-making process, the influence on the decision-making capacity of physicians and patients, the access to personal information, intellectual property issues, the balance of benefits and harms to patients, and the burden of a possible mistake in the simulation due to potential sources of bias leading to an incorrect definition of the algorithm.

Defining what the priorities are goes beyond the scope of this work and strongly depends on the specific contexts (social, economic, and technological). Considering the effectiveness of digital solutions in marginalized populations requires a greater effort, strongly linked both to the presence of the necessary digital infrastructure and the digital literacy of the target groups. In Europe, this could be carried out within the NextGenerationEU (NGEU) fund, a European initiative to provide financial support to all member states to recover from the adverse effects of the COVID-19 pandemic [71]. Recognizing that digitalization is a still open challenge, NGEU aims, among other things, to improve citizens’ digital skills and healthcare services through artificial intelligence and to fund training for Europe’s healthcare professionals to improve digital competencies. Given the industrialization of in silico medicine, continuous training should also involve the providers of the new technologies.

Artificial intelligence is one of the drivers of biomedical innovation. How the use of AI-powered systems, especially those of the latest generation, such as deep learning, to inform decisions affecting humans’ lives should satisfy the requirement of understanding has been widely discussed in Section 3. In recent years, the first ideas about the possibility of providing “explainability” have emerged and immediately become a hot topic (as in the Explainable AI (XAI) program): to create models that, combined with adequate explanatory techniques, allow end-users to understand the system [72]. A great deal of effort should be made to allow clinicians and practitioners to fully discuss the proposed interpretability methods rather than simply compare some accuracy indices. Recognizing that, in any case, the technical skills required for understanding the methods may not be available to physicians and end users, we must not forget the importance of careful and extensive validations of the methods on different populations as a tool for increasing confidence in methods themselves. In addition, more studies are needed that compare different methods, and poor results and inconsistencies should be highlighted as much as good ones.

To implement technology in healthcare, it is necessary to create a multi-stakeholder group that brings together experts from clinical/corporate/business and ICT backgrounds, organizations, and individuals engaged in all aspects of health. Stakeholders include end-users (such as patients and their organizations, caregivers) who could help identify issues impacting informed decisions, accessibility, and data protection, healthcare staff who can contribute to investigating changes in the decision-making process, health care managers and IT departments who can assess the implications for the planning of resources and infrastructure, and policy-makers who are interested in equity and distributive justice. This difficulty is strictly linked not only to the heterogeneity among stakeholders but also to: the asymmetric relationship concerning power, resources, and knowledge; involvement of experts in a long-term perspective of public administrators as opposed to the usual short-term perspective of technology release; definition of the cooperation framework. Establishing clear rules for stakeholder participation in HTAs that would balance all these issues could help foster trust and credibility for a better decision-making process.

Our analysis shows that the ongoing debate among decision-makers, developers, and stakeholders on the effects of CM&S needs both further discussions on several still-open issues, given the potential central role that this technology will play in healthcare processes in the near future, and the development of new methods in all the disciplines involved to deal with the challenge of technology implementation.

## Figures and Tables

**Table 1:** Social domain: practical examples of questions and suggested methods.

Issue	Specific Question	Suggested Methods						
		Systematic Assessment of Literature	Quantitative Data Generation		Qualitative Data Generation			Analysis of Technical Documentation
			Physicians	Citizens	Experts	Physicians	Citizens	
Cultural Issues: cultural resistance	Are there any prejudices about the effectiveness of the technology?	X			X	X	X	
Cultural Issues: level of expertise needed	Is there any evidence of the learning curve?	X	X					
	Is external training required before use?		X			X		X
Cultural Issues: explanations to patients	Are there good decision aids to support shared decision-making?	X			X	X	X	
	How involved do patients feel?						X	
	Do patients have the possibility of discussing with physicians how the model works, the potential resulting options, and the degree of reliability?					X	X	X
	Which is the minimum required level of digital literacy and health literacy?			X			X	X
Infrastructural issues	Which is the level of infrastructure needed, and what is the level of access for intended users?				X	X	X	X

**Table 2:** Ethical and legal domains: practical examples of questions and suggested methods.

Issue	Specific Question	Suggested Methods							
		Systematic Assessment of Literature	Quantitative Data Generation		Qualitative Data Generation			Analysis of Technical Documentation	Analysis of Regulations
			Physicians	Citizens	Experts	Physicians	Citizens		
Harms	Health and bodily harm *	X	X	X	X	X	X		
	Psychological harm *	X	X	X	X	X	X		
	Harms to society *	X			X				
Rights	Freedom of choice *	X					X		
	Patient right to autonomy *	X					X		
	Responsibility and accountability *	X			X		X	X	X
	Informed consent *	X			X		X	X	X
	Information Privacy (including harmful usage, security breach) *	X			X		X	X	X
Wellbeing	Health *	X	X	X		X	X		
	Social inclusion *	X	X	X	X		X		
Justice (distributive)	Nondiscrimination and equal treatment relative to age, gender, sexual orientation, social class, race, ethnicity, religion, disability *	X			X	X	X	X	
Technology aging	Are enough elements provided to compare the assessed technology with previous versions?							X	
Technology integration	Is standardization of data formatting considered?				X			X	X
Technology accessibility	Which measures are considered to guarantee equity in access?				X			X	
Marketing Authorization	Which implications for humans and animals?				X			X	X
	Are reference best practices adopted?				X			X	X
Intellectual property of innovations	(In case of technologies developed with public funds) Is there a mechanism of social compensation?							X	

* The Specific Question is: “Is there an issue in terms of [THE MARKED ELEMENT]?”

## Data Availability

Data is contained within the article.
